# Testing previous model predictions against new data on human papillomavirus vaccination program outcomes

**DOI:** 10.1186/1756-0500-7-109

**Published:** 2014-02-25

**Authors:** Megan A Smith, Karen Canfell

**Affiliations:** 1 Lowy Cancer Research Centre, Prince of Wales Clinical School, University of New South Wales, Sydney, NSW 2052, Australia; 2 School of Public Health, University of Sydney, Sydney, NSW 2006, Australia

**Keywords:** Human papillomavirus, HPV, Vaccination, Immunization program, Mathematical model, Australia

## Abstract

**Background:**

Vaccination against human papillomavirus (HPV), predominantly targeting young females, has been introduced in many countries. Decisions to implement programs, which have involved substantial investment by governments, have in part been based on findings from cost-effectiveness models. Now that vaccination programs have been in place for some years, it is becoming possible to observe their effects, and compare these with model effectiveness predictions made previously.

**Findings:**

Australia introduced a publicly-funded HPV vaccination program in 2007. Recently reported Australian data from a repeat cross-sectional survey showed a substantial (77%) fall in HPV16 prevalence in women aged 18–24 years in 2010–2011, compared to pre-vaccination levels. We have previously published model predictions for the population-wide reduction in incident HPV16 infections post-vaccination in Australia. We compared prior predictions from the same model (including the same assumed uptake rates) for the reduction in HPV16 prevalence in women aged 18–24 years by the end of 2010 with the observed data. Based on modelled vaccine uptake which is consistent with recent data on three-dose uptake (78% at 12–13 years; lower uptake in older catch-up age cohorts), we had predicted a 70% reduction in prevalence in 18–24 year old females by the end of 2010. Based on modelled vaccine uptake consistent with recent national data for two-dose coverage and similar to that reported by women in the cross-sectional study, we had predicted a 79% reduction.

**Conclusions:**

A close correspondence was observed between the prior model predictions and the recently reported findings on the rapid drop in HPV prevalence in Australia. Because broadly similar effectiveness predictions have been reported from other models used for cost-effectiveness predictions, this provides reassurance that the substantial public investment in HPV vaccination has been grounded in valid estimates of the effects of vaccination.

## Findings

### Background

Vaccination against human papillomavirus (HPV), predominantly targeting young females, has been introduced in many countries. Implementation of publicly-funded programs has involved substantial investment by governments. In Australia, for example, the government budgeted approximately A$580 million over the first five years of its National HPV Vaccination Program for females (2007–2011) [1,2]. In most affluent countries, decisions to implement HPV vaccination on a widespread basis have in part been made on the basis of findings from cost-effectiveness models [3]. The cost-effectiveness of HPV vaccination is critically dependent on its effectiveness in preventing HPV infection, the associated projected fall in population incidence and prevalence of infection, and thus prevention of the downstream sequelae [3]. These sequelae include the development of cervical precancerous abnormalities, their diagnosis and treatment, and the development of invasive cervical cancer, as well the development of anogenital warts and other types of HPV-associated cancer in the anogenital tract and head and neck in both females and males [4].

Now that vaccination programs have been in place for some years, it is becoming possible to observe their effects on health outcomes (both in the target population and also, due to herd immunity, in other populations), outside of the context of clinical trials. Australia implemented a National HPV Vaccination Program in 2007, with routine vaccination of 12–13 year old females and catch-up in females aged 13–26 years to 2009 with the quadrivalent vaccine (Gardasil®, Merck&Co., Whitehouse Station, NJ USA). The National HPV Vaccination Program is primarily delivered through schools, although missed doses can be obtained from primary care. The catch-up program was delivered through schools for school-aged girls, and through primary care for older (18–26 years) females or females who were no longer at school. Commencing from 2013, the National HPV Vaccination Program was extended to include routine vaccination of 12–13 year old males and a two year catch-up program for boys aged 14–15 years during 2013 and 2014. Australia offers unique opportunities to observe the early impacts of HPV vaccination, as its program commenced earlier and its catch-up component was more extensive than in most other countries. Post-vaccination reductions have already been documented in Australia in genital warts [5,6], high-grade cervical abnormalities [7,8], and most recently, HPV prevalence [9], in young women.

Tabrizi *et al.* recently reported a substantial fall in HPV prevalence in a repeat cross-sectional study in Australian women aged 18–24 years recruited during 2010–2011, compared to women of the same age recruited in 2005–2007, which was prior to the commencement of the vaccination program [9]. HPV16 prevalence was found to be 77% lower in the post-vaccine group than in the pre-vaccine group, and the authors found a significant reduction in the prevalence of vaccine-included HPV types (HPV16,18,6,11) after adjusting for other risk factors (adjusted OR = 0.16; 95%CI: 0.09-0.26) [9]. Self-reported vaccine uptake was high in the post-vaccination study sample, who were aged approximately 14–21 years in 2007 (86.4% one dose; 70.6% three doses), compared to the population uptake estimated for that age group based on data held by the National HPV Vaccination Program Register (NHPVR) (estimated 72.4% one dose; 52.2% three doses) [9-11]. The actual difference in uptake may be smaller than these figures suggest, however, as related work in Australia has suggested self-reported vaccination status tends to slightly over-estimate uptake, and conversely, national coverage data from the NHPVR may be under-reported [9,11]. Under-reporting, particularly of doses delivered through primary care to females no longer in school or females who missed doses at school, is thought to be likely as notification of these doses to the NHVPR was voluntary (although a small incentive payment of A$ 6 per dose notified was available). Also, because of the very rapid roll-out of the vaccination program, the NHVPR did not commence operations until June 2008, the year after the program commenced, and providers needed to retain data for doses administered prior to this time [11]. Under-reporting to the NHVPR is also suggested by data from earlier surveys, by discrepancies between data for doses distributed versus doses notified, and by wide variations in reported uptake between states, with higher uptake reported in states having centralised reporting mechanisms in place even though the vaccination program was promoted at a national level [11].

We have previously published model predictions for the reduction in incident HPV16 infections post-vaccination in Australia, across the female population [12,13]. The aim of this study was to compare these previous model predictions with the findings of Tabrizi *et al.*, which required specific examination of the prior model predictions for HPV prevalence in the specific age group and at the specific timepoint reported by Tabrizi *et al.*[9]. We also compared the original estimates of vaccine uptake used in the previous analysis with observed uptake in Australia, and in relation to the women in Tabrizi *et al.*[9,12].

## Methods

In line with methods used by several other groups [3], we used a dynamic model of HPV transmission and vaccination. This is the most comprehensive method of estimating the effectiveness, and hence the most comprehensive method of estimating the cost-effectiveness, of HPV vaccination, and takes into account herd immunity, the potential for prior HPV exposure in catch-up cohorts, and detailed and differing coverage among the catch-up cohorts [3]. This model has been described in detail previously [12-14]. Briefly, the model simulates the potential transmission of HPV16 during heterosexual partnerships in a population which is closed and stratified by sex, age and level of sexual activity. Model parameters were obtained via literature reviews and fitting to observed data [12,13]. Prior to modelling the effect of vaccination, the model was calibrated to cross-sectional age-specific survey data on HPV prevalence in sexually active women, from an initial pre-vaccination cross-sectional survey in women presenting for cervical screening in Australia (WHINURS) [12,14,15]. The pre-vaccine sample in Tabrizi *et al.* also comprised the subset of women from WHINURS who were 18–24 years of age at the time of recruitment.

To compare model predictions with the findings of Tabrizi *et al.*[9], we extracted the model-predicted prevalence at the end of 2010 (around the midpoint of the recruitment period for Tabrizi *et al.*[9]) among women aged 18–24 years during the period 2010–2011 (the “comparison population”). Women in the model who were aged 24 in 2010 or aged 18 in 2011 were given a lower (50%) weighting in the model-based prevalence calculations than women who were aged 18–24 years in both 2010 and 2011, since these women were not eligible for recruitment over the entire period of the study. This group of women were offered vaccination in Australia during 2007–2009 as 14–21 year olds.

We had modelled uptake in terms of effective vaccination; initially we had assumed three vaccine doses would be needed to achieve the efficacy levels observed in clinical trials [12]. However, recent data suggest the possibility that two-dose efficacy may be comparable to three-dose efficacy [16,17]; thus, modelled predictions based on coverage which is comparable to two-dose coverage rates achieved may also be relevant.

Vaccine uptake scenarios (and all other model parameters) were unchanged compared to our previous analysis [12]. The early estimates of uptake which were incorporated in the original modelled analysis have now been compared to recently reported data on achieved uptake in Australia [10,11,18] and to that reported by the specific group of women reported on by Tabrizi *et al.*[9]. Table 1 shows how uptake in the comparison population (females aged 18–24 years in 2010–2011) in each of the scenarios modelled compares to achieved uptake, both in the Australian female population that age, and in the specific group of women reported on by Tabrizi *et al.*[9]. Full details of the age-specific uptake rates we had assumed are reproduced in Additional file 1: Table S1 [12]. In the main modelled scenario, effective vaccine uptake in the comparison population of females was 57.7% (feasible range: 48.8-68.6%). This modelled uptake is broadly consistent with recent data on three-dose uptake in Australia in females aged 18–24 years in 2010–2011 (57.7% modelled vs 52.2% estimated from reported data [9-11,19]), but lower than that reported by women in Tabrizi *et al.* (70.6%; although this was potentially over-reported) [9]. Uptake in the main scenario we modelled is also broadly consistent with recent estimates of three-dose uptake in younger females (aged less than 18 years in 2007), but higher than reported three-dose uptake in females vaccinated in the primary-care-based catch-up program (aged 18 or older in 2007) (Additional file 1: Table S1), although potential under-reporting to the NHPVR should be taken into account [9,11]. In sensitivity analysis we had also modelled a wide range of possible uptake scenarios [12]. The higher end of the feasible range we had examined (68.6% in the comparison population) is consistent with recent data for two-dose coverage in Australia in females aged 18–24 years in 2010–2011 when potential under-reporting to the NHPVR is taken into account (estimated 63.6-67.7%) [9,11] and also similar to that reported by women in Tabrizi *et al.* (70.6%) [9] (Table 1). The higher end of the feasible range we had modelled is also broadly consistent with reported national two dose uptake in younger females (aged less than 18 years in 2007), but is higher than reported two-dose uptake in females vaccinated in the primary-care-based catch-up program (aged 18 or older in 2007) (Additional file 1: Table S1), although it is possible that this two-dose data was also under-reported to the NHVPR [11,20]. The lower end of the feasible range we had modelled can now be seen as lower than reported three-dose uptake (48.8% modelled vs 52.2% estimated from reported data [9-11,19]), particularly when potential under-reporting to the NHPVR is taken into account, and substantially lower than that reported by women in Tabrizi *et al.* (Table 1, Additional file 1: Table S1) [9].

**Table 1:** **Uptake in the comparison population**^**a**^**: comparison of uptake in females included in Tabrizi et al. [**[9]**], Australian population, and modelled population**

**Vaccine uptake**	**Repeat cross-sectional sample in Tabrizi *et al. *** [9]	**Australian population**	**Modelled population**
	**(females 18–24 years)**^**a**^	**(females 18–24 years)**^**a,b**^	**(females 18–24 years)**^**a**^
> = 1 dose	85.6%^c^	72.4%	na^d^
> = 2 doses	[not reported]	63.6%	na^d^
3 doses	70.6%^c^	52.2%	na^d^
Effectively vaccinated^d^			57.7%^e^ (48.8 - 68.6%)^f^

^a^Females aged 18–24 years in 2010–2011^b^ calculated from published data from NHVPR; potentially under-reported [10,11]^c^ self-reported uptake^d^ Not applicable, as doses are not explicitly modelled; uptake in the model reflects the proportion who are effectively vaccinated ie doses needed to achieve the efficacy levels observed in clinical trials^e^ Main scenario used in original analysis, based on early estimates of coverage^f^ Feasible range used in original analysis, based on early estimates of coverage.

## Results

In the main scenario examined (modelled uptake in the comparison population 57.7%; broadly comparable to observed three-dose uptake in Australian females that age (52.2-55.1%), but lower than self-reported uptake in Tabrizi *et al.* (70.6%) [9]), we had predicted a 41% reduction in incident HPV16 infections in females across all ages by the beginning of 2010 [12]. When we examined predictions specifically for females aged 18–24 years from the same uptake scenario, we found a 60% reduction in incident infections in 18–24 year old females by the beginning of 2010. This in turn corresponded to a 63% reduction in HPV16 prevalence in females in this age group. Considering model predictions specifically for a time point and group of females broadly equivalent to those included in Tabrizi *et al.* (females aged 18–24 years old at some point during the recruitment period (2010–2011)), the model predicted a reduction in HPV16 prevalence of 70% by the end of 2010 (close to the midpoint of recruitment in Tabrizi *et al.*) [9]. Based on the higher end of the modelled feasible range (assumed uptake in the comparison population 68.6%; broadly consistent with two-dose data for that group (63.6-67.7%) [20]; similar to three-dose uptake reported by women in Tabrizi *et al.* (70.6%) [9]), we predicted a 79% reduction in 18–24 year olds by the end of 2010. Based on the lower end of the feasible range (assumed uptake in the comparison population 48.8%; lower than three-dose uptake based on NHVPR data (52.2-55.1%); considerably lower than the self-reported three-dose uptake rates in Tabrizi *et al.* (70.6%) [9]), we predicted a 64% reduction in prevalence in 18–24 year old females by the end of 2010.

## Discussion

This predicted 64%-79% fall in HPV16 infections in 18–24 year old females by the end of 2010 accords well with the data from Tabrizi *et al.*, who reported a 77% reduction in HPV16 prevalence, [9] especially in light of the higher self-reported uptake in the study group, and also the fact that the upper end of our originally modelled feasible range for uptake may better reflect observed uptake in the population, under-reporting to the NHVPR, and the potential for high two-dose efficacy.

While our model incorporated the best estimates of uptake available at the time, and attempted to encompass a broad feasible range, there are now more data available on both uptake achieved by the program and the efficacy of two versus three doses of quadrivalent vaccine. The uptake we assumed for our original predictions [12] is generally consistent with reported three-dose uptake after accounting for under-reporting to the NHVPR in the older catch-up cohorts [10,11,20]. The upper end of the feasible range examined is broadly consistent with observed two-dose data, in the context of under-reporting to the NHVPR. However, based on the latest data on uptake, it now seems that the lowest end of the feasible uptake range we had assumed (48.8% for the comparison population; 70% at age 12, and ranging from 70% at age 13 to 15% at age 26 years) is below observed three-dose uptake, and so our original predictions based on this end of the range may be less relevant, especially in the context of under-reporting to the NHVPR and two-dose efficacy being high [16,17]. As a result of these observations, our predicted reductions in HPV16 prevalence in the range of 70-79% now seem the most relevant for the national Australian population, although even they may not fully account for under-reporting to the NHVPR in the context of high two-dose efficacy. They may also underestimate the effects seen in a group like those included in Tabrizi *et al.*, where three-dose uptake was potentially somewhat higher than average for other females of the same age [9]. Two-dose uptake was not reported for this group, but since it was almost certainly higher than three-dose uptake, it was thus higher than in any scenario we modelled. Consistent with this, the reduction in the prevalence of vaccine-included types observed by Tabrizi *et al.* was substantial, after adjusting for differences in age and hormonal contraceptive use in the pre- and post-vaccination study samples [9].

Models of HPV vaccination typically assume high vaccine efficacy in the base case [3], based on the “per-protocol” results from clinical trials which reflect efficacy in HPV-naïve individuals. In order to make predictions about program effectiveness, and thus cost-effectiveness, models use this information in conjunction with other epidemiological and behavioural data, in order to take into account the interaction of other complex factors such as the natural history of HPV infection, and sexual behaviour and contact patterns. The outcomes of real world programs which are observable in the short term, predominantly reflecting outcomes in older catch- cohorts, will be particularly influenced by prior exposure to HPV and thus the complex interaction of these factors. Taking these interactions into account is necessary to translate efficacy data from clinical trials into predictions of effectiveness in the real world, but can make models complex and less transparent to policymakers and clinicians. This makes it especially important to check prior model predictions when the data become available to do so; however this is not routinely done. Our model findings for a rapid drop in HPV incidence and prevalence in young women and predictions of substantial reductions over the longer term have been broadly mirrored by findings from other groups using dynamic models where the impact on HPV infections have been reported [21-23]. Findings from these and other models have been influential in cost-effectiveness decisions in affluent countries [3].

The joint task force of the International Society for Pharmacoeconomics and Outcomes Research (ISPOR) and the Society for Medical Decision Making recommended as best practice for medical decision-making models that model predictions of future events should be tested against observed data, when these data subsequently become available [24]. This is important, in order to test the reliability of model-based predictions, and justify the substantial public investment made on the basis of those predictions. To our knowledge, this is the first study which has compared prior model predictions of HPV vaccination program impacts to subsequently observed program outcomes, even though policy decisions around HPV vaccination have generally been made on the basis of such models. Australia is among the first countries in the world where this has been possible, as Tabrizi *et al.* noted that, as far as they were aware, their findings “were the first genoprevalence-based evidence of the protective effect of HPV vaccination outside the setting of a clinical trial”[9].

## Conclusions

The investments in HPV vaccination programs around the world have been substantial. Such investments in affluent countries have been made, in part, on the basis of predictions from cost-effectiveness models. However, prior model predictions for vaccine effectiveness in a 'real world’ population have not previously been compared to observed data after the vaccination program has been implemented.

The close correspondence between these prior model predictions and the recently reported findings on the rapid drop in HPV prevalence rates in Australia [9] provides reassurance that the substantial public investment in HPV vaccination has been grounded in valid estimates of the effects of vaccination programs.

## Abbreviations

HPV: Human papillomavirus; NHVPR: National HPV vaccination program register.

## Competing interests

MS: has no commercial or other association that might pose a conflict of interest. KC: declares that she is involved as a principal investigator in a new trial of primary HPV screening in Australia, which is supported by Roche Molecular Systems and Ventana, CA, USA. KC receives salary support from the NHMRC Australia (CDF1007994).

## Authors’ contributions

MS: Designed the study; analysed and interpreted the data; drafted the manuscript. KC: Conceived and designed the study; helped draft the manuscript. Both authors read and approved the final manuscript.

## Supplementary Material

Additional file 1: Table S1 Age-specific vaccine uptake in females in modelled scenarios versus reported uptake in Australian female population. Click here for file
