# Supplementary material for: Testing previous model predictions against new data on human papillomavirus vaccination program outcomes
Source: BMC Res Notes. 2014 Feb 25;7:109. doi: 10.1186/1756-0500-7-109 (PMC3938033; doi:10.1186/1756-0500-7-109)
Supplement: Additional file 1: Table S1 — Age-specific vaccine uptake in females in modelled scenarios versus reported uptake in Australian female population. [file 1756-0500-7-109-S1.doc]

**Additional file 1: Table S1 – Age-specific v**accine uptake in females in modelled scenarios versus reported uptake in Australian female population

|  | Original modelled percentage of females effectively vaccinated | | Recent national data on uptake in Australian population *(adjusted for under-reporting)** | | | |
| --- | --- | --- | --- | --- | --- | --- |
| Age (in 2007) | Main scenario | *Feasible range* | Three doses | | Two doses | |
| <12 (ongoing)† | 78 | *70-80* | 72 - 73† |  |  | |
| 12 | 75 | *70-80* | 74 |  | 80 |  |
| 13 | 74 | *70-80* | 73 |  | 80 |  |
| 14 | 71 | *70-80* | 71 |  | 79 |  |
| 15 | 71 | *70-80* | 72 |  | 80 |  |
| 16 | 67.5 | *60-75* | 69 |  | 77 |  |
| 17 | 67.5 | *60-75* | 62 |  | 72 |  |
| 18 | 50 | *40-60* | 41 | *(47.5)* | 55 | *(63.8)* |
| 19 | 45 | *30-60* | 36 | *(41.8)* | 50 | *(58.0)* |
| 20 | 45 | *30-60* | 34 | *(39.4)* | 49 | *(56.8)* |
| 21 | 45 | *30-60* | 33 | *(38.3)* | 47 | *(54.5)* |
| 22 | 45 | *30-60* | 32 | *(37.1)* | 46 | *(53.3)* |
| 23 | 45 | *30-60* | 32 | *(37.1)* | 46 | *(53.3)* |
| 24 | 45 | *30-60* | 32 | *(37.1)* | 45 | *(52.2)* |
| 25 | 40 | *25-55* | 28 | *(32.5)* | 40 | *(46.4)* |
| 26 | 25 | *15-35* | 17 | *(19.7)* | 24 | *(27.8)* |

* Not all doses delivered in the catch-up program through primary care were recorded on the NHVPR. Adjusted for under-reporting using validation data held by the NHVPR (personal communication: Dr Julia Brotherton, Medical Director, National HPV Vaccination Program Register, Melbourne, Australia) † Cohorts offered vaccination as 12 year olds in 2008 or later. To date three-dose uptake data only is available, for the 2008 and 2009 cohorts only.
